# Enhanced Performance of nano-Bi_2_WO_6_-Graphene as Pseudocapacitor Electrodes by Charge Transfer Channel

**DOI:** 10.1038/srep08624

**Published:** 2015-02-27

**Authors:** Jun Zhang, Pengliang Liu, Yupeng Zhang, Guolong Xu, Zhengda Lu, Xiyu Wang, Yan Wang, Lingxia Yang, Xi Tao, Hongbo Wang, Erpan Zhang, Junhua Xi, Zhenguo Ji

**Affiliations:** 1 College of Materials and Environmental Engineering, Hangzhou Dianzi University, Hangzhou, 310018, People's Republic of China; 2 State Key Lab of Silicon Materials, Zhejiang University, Hangzhou, 310018, People's Republic of China; 3 College of Automation, Hangzhou Dianzi University, Hangzhou, 310018, People's Republic of China; 4 Department of Materials Engineering, Monash University, Victoria, 3800, Australia

## Abstract

The nano-Bi_2_WO_6_/reduced graphene oxide composite obtained by a simple hydrothermal reaction demonstrates a larger specific capacitance of 922 F/g at a charge and discharge currents of 3 A/g with longer cycle life. The As comparison, pristine Bi_2_WO_6_ nanoparticles have poor specific capacitance of 574 F/g at a charge and discharge currents of 2 A/g with weak cycle life. Though analyzing the Cyclic voltammetry curves, it is found that there are two oxidation reaction occurring in the materials: oxidation of Bi (III) to Bi (IV) and Bi (III) to Bi (V). The oxidation of Bi (III) to Bi (IV) is reversible while Bi (III) to Bi (V) will cause nonreversible destroy on structure. In this nano-Bi_2_WO_6_/reduced graphene oxide composite, graphene with well conductivity will enhance the electrically conducting as charge transfer channel, so that electrons will be transfer much faster in oxidation and most Bi (III) is oxidized to be Bi (IV) which ensure larger specific capacitance and long cycle life. This nano-Bi_2_WO_6_/reduced graphene oxide composite has application prospect in high-performance pseudo-capacitors.

Energy storage and conversion from semiconductor and alternative energy sources have stimulated a large of research due to the urgency demand for energy. Photovoltaic cells, batteries, fuel cells, and supercapacitors are mainly Energy storage and conversion devises^1^,^2^,^3^,^4^,^5^. Supercapacitors are considered as a ideal short-term energy storage due to fast charge-discharge, long cycle life, high power performance and low maintenance cost^6^,^7^,^8^,^9^,^10^,^11^. For using as primary power sources as batteries, it is highly desirable to increase the energy density. The energy density of traditional double layer capacitors can hardly increase. Some redox during charge-discharge of pseudo-capacitive materials such as hydroxides^12^,^13^,^14^, oxides^15^,^16^,^17^,^18^,^19^,^20^,^21^ and polymers^22^,^23^,^24^ can sharply increase the specific capacitance and high energy density. However, the electrical conductivity this pseudo-capacitive materials are too low to support high rate electron transport.

Graphene is a two-dimensional material with an ideal single-atom-thick or several layers of limited structure, and it has attracted much attention recently due to its many potential applications in electronic, optical, catalytic and energy fields^25^,^26^,^27^,^28^. Due to the high surface area, good electrical conductivity, light weight, high flexibility and mechanical strength, graphene is a ideal substrate as charge transfer channel for photoelectrochemistry^29^,^30^,^31^, or energy storage^32^,^33^,^34^,^35^,^36^. Pt^32^, metal oxides^33^,^34^,^35^ and polymers^36^ were coupled with reduced graphene oxide or highly conducting graphene sheets to improve performance applied in Li-ion battery and supercapacitor. Bi_2_WO_6_ is a kind of Aurivillius phase oxide with a layered structure of the perovskite layer (WO_4_)^2−^ lies between (Bi_2_O_2_)^2+^ layers^37^. Bi_2_WO_6_ has excellent physical and chemical properties such as oxide anion conducting, a nonlinear dielectric susceptibility, ferroelectric piezoelectricity, pyroelectricity, catalytic behavior and luminescent properties^38^,^39^,^40^,^41^. Bi_2_WO_6_-grphene has been prepared and the photocatalytic performance was shown^42^,^43^,^44^. However, Bi_2_WO_6_-grphene as pseudocapacitor electrodes has not been reported.

In this work, we reported a simple method to obtain nano-Bi_2_WO_6_-graphene composite which demonstrate larger specific capacitance, higher energy density and longer cycle life. As comparison, plain nano-Bi_2_WO_6_ has poor specific capacitance and weak cycle life due to the low electric conduction resulted in most Bi (III) will be over oxidized to be Bi (V) which causes nonreversible destroy on structure. In this nano-Bi_2_WO_6_-graphene, graphene will render the electrically conducting as charge transfer channel. In this case, Bi (III) is more inclined to be Bi (IV) in charging instead of Bi (V) which ensure the material has long cycle life.

## Results

**Figure 1:**
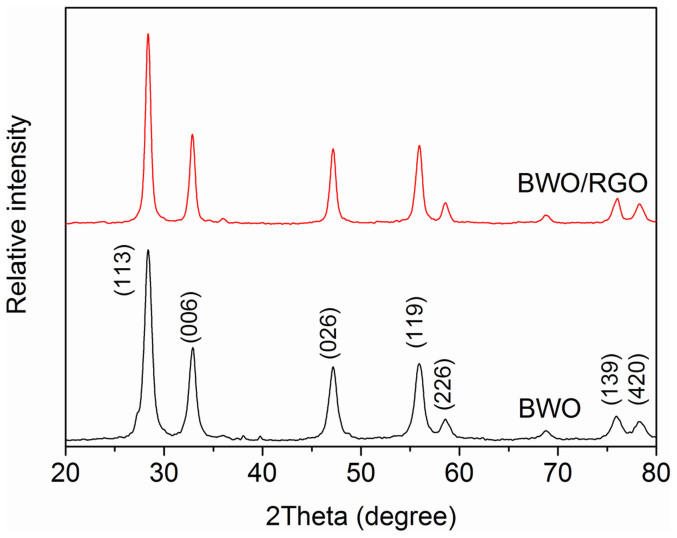
XRD patterns of BWO and BWO/RGO.

The BWO was obtained by a hydrothermal route while the BWO/RGO was synthesized via a two-step method with two hydrothermal progress. The crystal structure of BWO and BWO/RGO were studied by XRD analysis in Figure 1. In the patterns, all peaks demonstrate sharp and well defined shape corresponding to the Bi_2_WO_6_ phase with orthorhombic russellite structure. The lattice parameter values deduced from the XRD peaks are in concordant with the standard JCPDS data file no 73-2020. No other matter is found confirmed the hydrothermal reaction occurred completely. Moreover, except there is little enhancement of crystallinity (about 10%), two XRD patterns of the BWO and BWO/RGO are almost the same which indicates that although under an extra hydrothermal progress, the BWO nanoparticles in the BWO/RGO composites have less change compared with pristine BWO. It seems that when exceeding 24 h, extra time for hydrothermal reaction has little influence on the structure of BWO.

**Figure 2:**
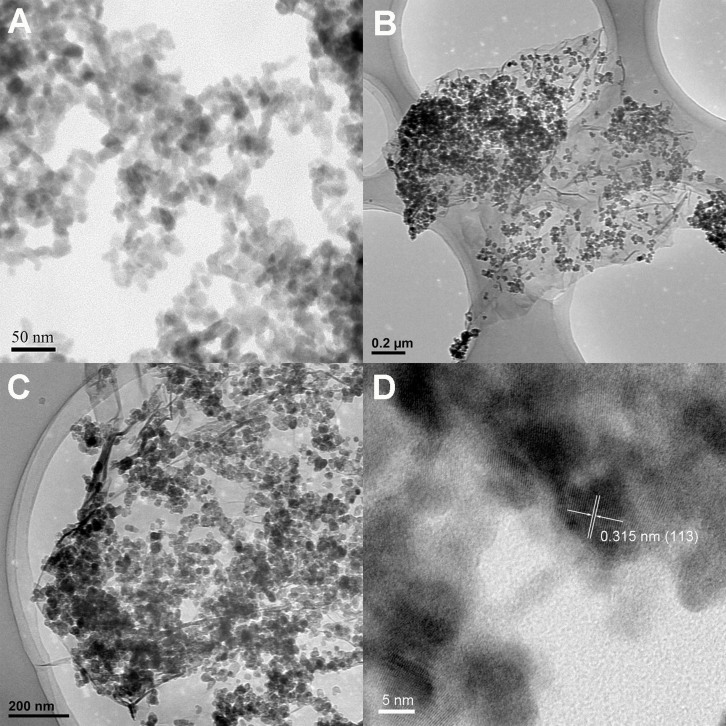
(A) TEM image of BWO; (B), (C) low resolution TEM images and (D) high resolution HRTEM image of BWO/RGO.

The TEM and HRTEM images of BWO and BWO/RGO are shown in Figure 2. The typical morphology of the BWO is found to be spherical in shape and uniform size distribution of nanoparticles (Figure 2A). The maximum number of BWO particles are found to be around 20 nm. The uniform BWO nanoparticles diffuse well on the graphene (Figure 2B and C) which can lead to a well contact between BWO and graphene. The results reveal that addition of graphene would not change the structure and morphology of the BWO nanoparticles. In the high-resolution image (Figure 2D), the lattice spacing of 0.315 nm can corresponding to the (113) plane of Bi_2_WO_6_.

As compared, another composite was obtained by a hydrothermal reaction between reduced graphene oxide (RGO) and BWO nanoparticles. As Figure S1 shown, little BWO nanoparticles composited on the surface of graphene. It seems the composite progress and the contact between BWO and graphene were promoted by the reduction of GO during the hydrothermal reaction. Raman spectra can further confirm the reduction of GO. Figure S2 illustrates Raman spectra of the GO synthesized chemical exfoliation and RGO from hydrothermal treatment. It is known that there are two Raman peaks can be observed from GO and RGO, that is, G peak at 1570 cm^−1^, D peak at 1360 cm^−1^. In general, the value of I_d_/I_g_ ratio reflects the surface defects density of graphene, which also give an information about the degree of reduction. From Figure S2, we can clearly see that more surface defects exist in the GO (I_d_/I_g_ = 1.08) than RGO (Id/Ig = 0.91). The results could confirm that the GO was reduced greatly after hydrothermal treatment.

**Figure 3:**
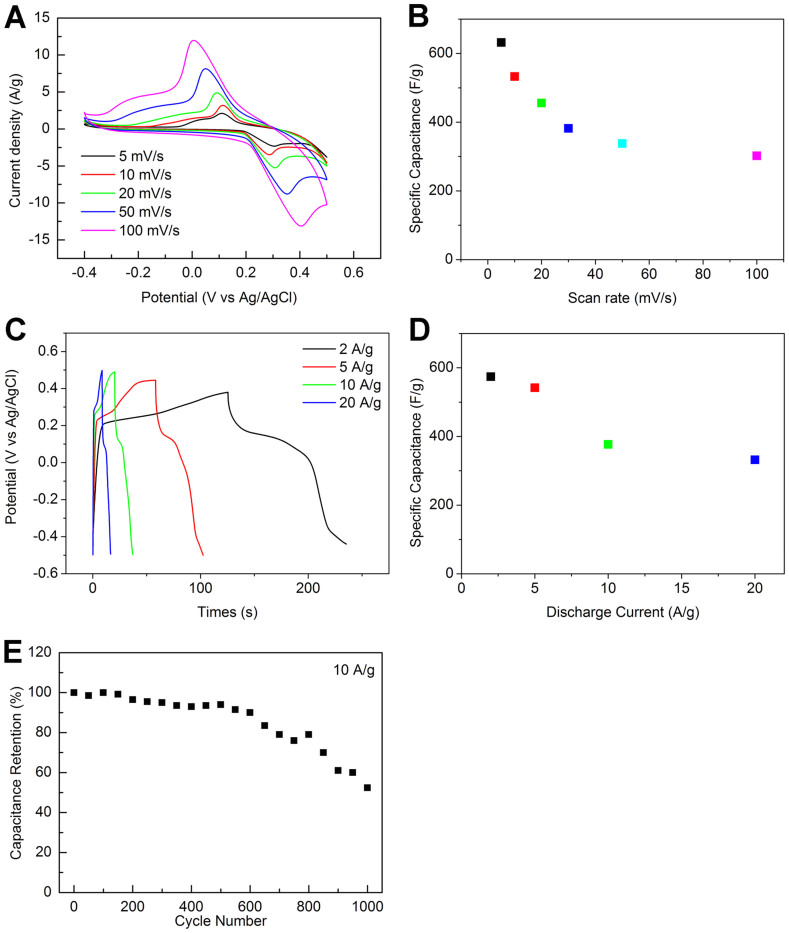
Electrochemical characterizations of BWO: (A) CV curves of BWO at various scan rates, (B) average specific capacitance of BWO at various scan rates; (C) galvanostatic discharge curves of BWO at various discharge current densities, (D) average specific capacitance of BWO at various discharge current densities; (E) average specific capacitance versus cycle number of BWO at a galvanostatic charge and discharge current density of 10 A/g.

Cyclic voltammetry (CV) is the most important measurement in electrochemistry which provides the qualitative information regarding the electrochemical processes that takes place in the material. The CV curves of the BWO at various scan rates are shown in Figure 3A. There is a pair of redox peaks observed for Bi_2_WO_6_ in the curves which indicates the pseudo capacitive behavior and these redox peaks are due to the oxidation and reduction reactions occurred in the material. The background signal due to the Ni foam was negligible^14^. The average specific capacitance of the BWO was calculated to be 632 F/g (based on mass of BWO) at a scan rate of 5 mV/s which is similar with other reports (Figure 3B)^45^, and 302 F/g at a high scan rate of 100 mV/s, about 48% of that at 5 mV/s. Figure 3C shows galvanostatic discharge curves of the BWO at various current densities. The BWO showed a specific capacitance of 574 F/g (based on mass of BWO) at a charge and discharge current density of 2 A/g (Figure 3D). When the charge and discharge current density was raised to be 20 A/g, the specific capacitance was only 332 F/g, about 58% of that at 2 A/g. Moreover, the columbic efficiency fell obviously when the cycle index of charge and discharge exceeded about 500 and was only 50% when the cycle index reach 1000. The results indicate that BWO should be improved for applying in supercapacitor.

**Figure 4:**
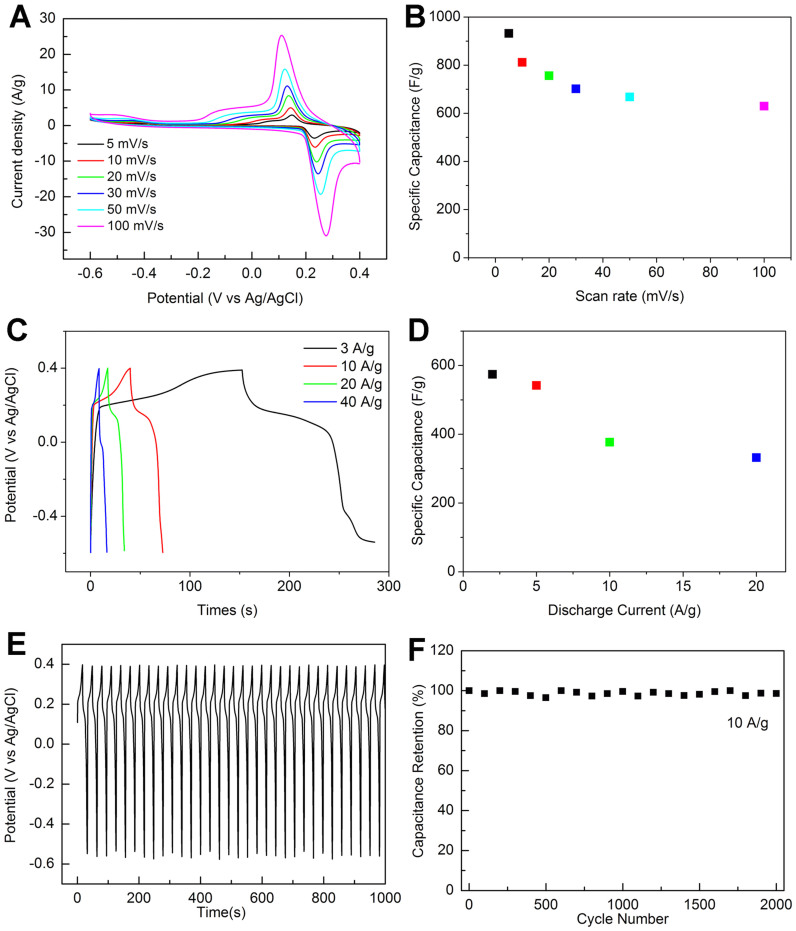
Electrochemical characterizations of BWO/RGO: (A) CV curves of BWO/RGO at various scan rates, (B) average specific capacitance of BWO/RGO at various scan rates; (C) galvanostatic discharge curves of BWO/RGO at various discharge current densities, (D) average specific capacitance of BWO/RGO at various discharge current densities; (E) galvanostatic charge and discharge curves of BWO/RGO at a current density of 10 A/g, (F) average specific capacitance versus cycle number of BWO/RGO at a galvanostatic charge and discharge current density of 10 A/g.

The BWO/RGO composites were carried out similar electrochemical measurements. In the CV curves (Figure 4A), the average specific capacitance of the BWO/RGO was improved to be 932 F/g (based on mass of BWO/RGO) at a scan rate of 5 mV/s (Figure 4B), even 630 F/g at a high scan rate of 100 mV/s, about 68% of that at 5 mV/s. As graphene has high surface area and good double layer capacitance^46^,^47^, the contribution of RGO to the total capacitance can not be ignored. The CV curves of RGO are shown as Figure S3. The double layer capacitance (about 150 F/g) of RGO was almost equal to the above one. Combining the CV curves of RGO/BWO, the contribution of RGO to the total capacitance could be calculated to be about 128 F/g. In the galvanostatic discharge curves of the BWO/RGO at various current densities in Figure 4C, the BWO/RGO showed a specific capacitance of 922 F/g (based on mass of BWO/RGO) at a charge and discharge current density of 3 A/g (Figure 4D). When the charge and discharge current density was raised to be 40 A/g, the specific capacitance was 663 F/g, about 72% of that at 3 A/g. Importantly, the columbic efficiency was nearly 100% for each cycle of charge and discharge (Figure 4E). There was no obvious capacitance decrease observed over 2000 cycles of charge and discharge at a current density of 10 A/g (Figure 4F). The results reveal the high specific capacitance and remarkable rate capability of the nano-Bi_2_WO_6_/reduced graphene oxide composite material for high-performance pseudo-capacitors.

**Figure 5:**
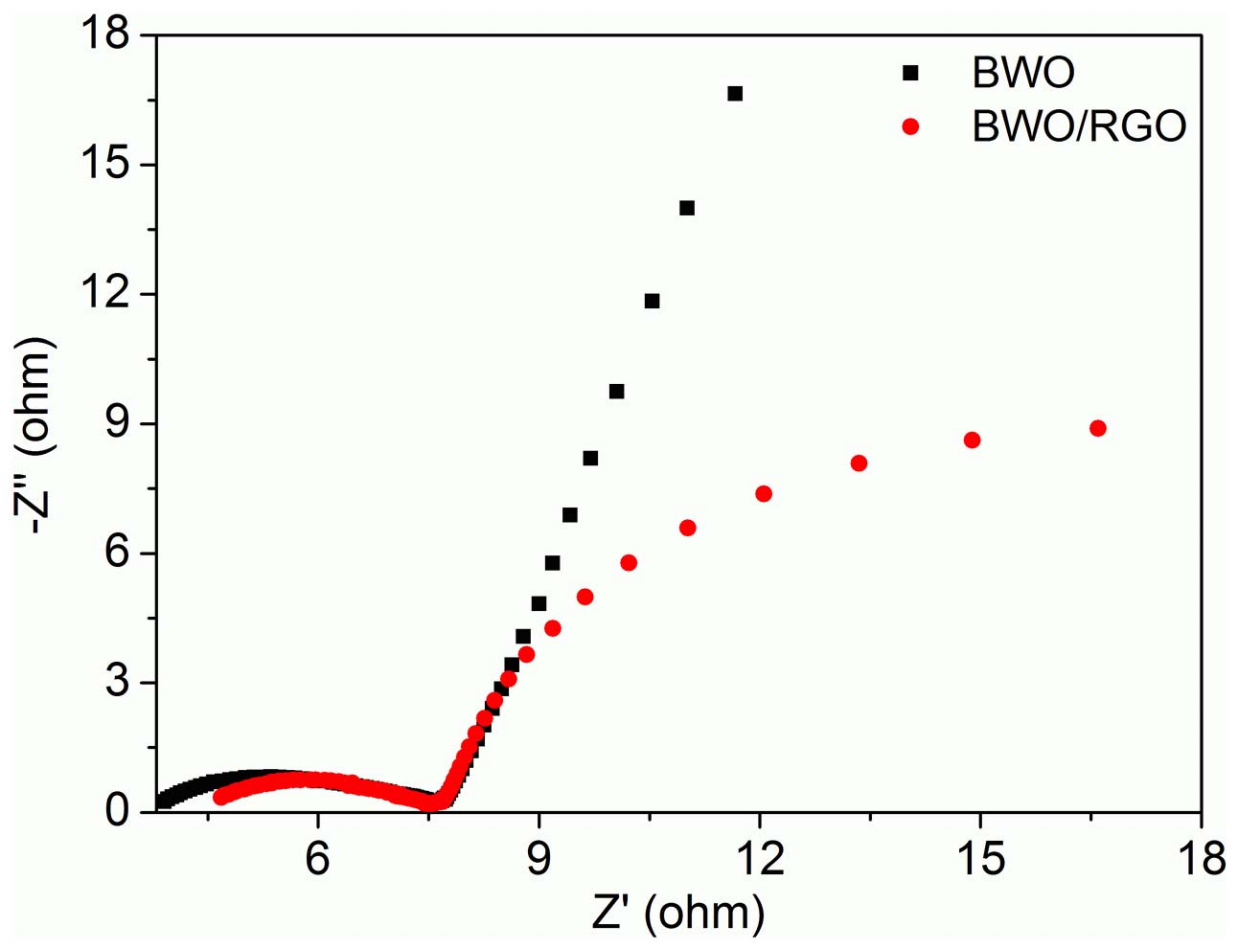
Electrochemical impedance spectroscopy (EIS) Nynquist plots of BWO and BWO/RGO electrodes.

The BWO/RGO exhibits excellent electrochemical characteristics and high cycling stability, making that potentially useful for high performance supercapacitor material. In same CV or galvanostatic method in three-electrode systems, the BWO/RGO composites show higher stable specific capacitance at higher charge/discharge rates than pristine BWO nanoparticles. There are several features lead to higher capacity and faster energy storage and releasing of BWO/RGO. First, the BWO nanoparticles are directly grown and anchored on reduced graphene oxide sheets after the hydrothermal reaction. The interactions between BWO nanoparticles and RGO could be both covalent chemical bonding and van der Waals interactions. This intimate binding offers facile electron transport between individual nanoparticles and RGO. Although BWO nanoparticles are electrically insulating, the RGO is well conductor and can transfer the electrons of BWO in redox which is key to both high specific capacitance and rate capability of the BWO/RGO. Most of the BWO nanoparticles in the system are electrochemically active through the graphene network. Rapid charge transport from BWO nanoparticles to the underlying RGO provides fast redox reactions. The better conductivity of BWO/RGO can be confirmed by the Electrochemical impedance spectroscopy (EIS) (Figure 5). In the Nynquist plots of EIS, the BWO/RGO electrode has a smaller frequency semicircle compared with BWO electrode which meanings the resistance of BWO/RGO electrode is lower.

**Figure 6:**
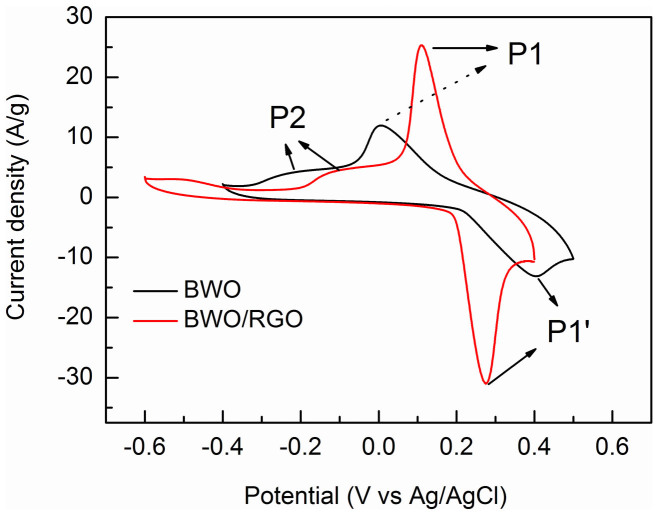
A comparison of CV curves of BWO and BWO/RGO at a scan rate of 100 mV/s.

**Figure 7:**
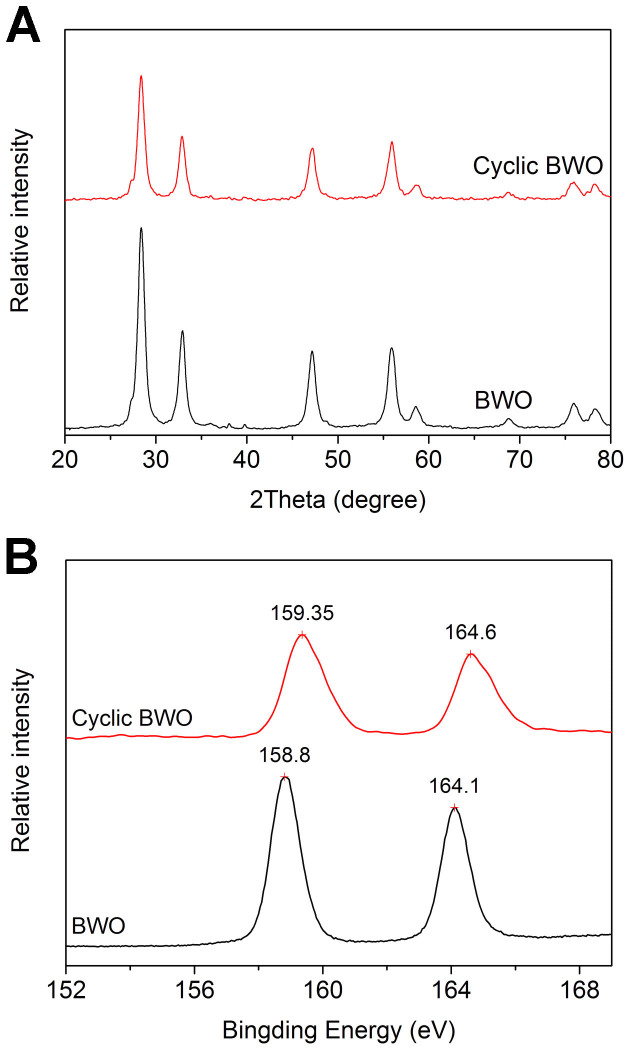
(A) XRD patterns and (B) XPS spectra of BWO and cyclic BWO.

The decline of columbic efficiency for BWO in the cycle of charge and discharge can be attributed to the irreversible oxidation in the redox reactions. A comparison of CV curves for BWO and BWO/RGO is shown in Figure 6. There are two oxidation peaks due to the oxidation of Bi (III) to Bi (IV) (labeled as P1) and Bi (III) to Bi (V) (labeled as P2) respectively, while only one reduction peak due to the reduction of Bi (IV) to Bi (III) (labeled as P1'). So that the oxidation of Bi (III) to Bi (IV) is reversible while oxidation of Bi (III) to Bi (V) is irreversible which results in the decline of columbic efficiency in the cycle of charge and discharge. The intensity of P2 peak of BWO and BWO/RGO are similar while the intensity of P1 peak of BWO/RGO is higher than that of BWO. Compared with BWO, the intensity ratio of P1/P2 of BWO/RGO is much higher. The result illustrates that in BWO/RGO, more Bi (III) is oxidized to Bi(IV) leads to a much high cycling stability. When graphene is absent, in oxidizing reaction, electrons are likely to gather in BWO nanoparticles due to the weak electrical conductivity which causes over oxidation of Bi (III) to Bi (V). As RGO is well conductor, the electrons will be transfer timely by the underlying graphene with less aggregation. To study the chemical state and structure changes after electrochemistry tests, the pristine BWO and cyclic BWO after 1000 cycles were detected by XRD and XPS (Figure 7). After 1000 cycles. the crystalline of cyclic BWO became weak means there is some damage on the crystal structure (Figure 7A). In the XPS spectra of BWO (Figure 7B), the binding energy at 158.8 eV and 164.1 eV belong to the Bi (III) 4f_7/2_ and 4f_5/2_ states^48^. The two peaks of Bi show a shift of about 0.5 eV toward higher banding energy. As higher binding energy meanings higher valence state, and combining with the P2 peak in CV curve (Figure 6), it can be confirmed that during electrochemistry progress, more Bi (III) is over-oxidized to Bi(V). Taken together, it is high important to use high electrical conductivity graphene as grown substrate to produce advanced nanocrystal/graphene composite materials for energy application.

## Discussion

In this work, we reported a simple method to obtain nano-Bi_2_WO_6_/reduced graphene oxide composite which demonstrate larger specific capacitance, higher energy density and longer cycle life. As comparison, pristine Bi_2_WO_6_ nanoparticles have poor specific capacitance and weak cycle life due to the low electric conduction and over oxidation of Bi (III) to Bi (V) which causes nonreversible destroy on structure. In this nano-Bi_2_WO_6_/reduced graphene oxide composite, graphene with well conductivity will enhance the electrically conducting as charge transfer channel. In this composite, electrons will be transfer timely in oxidation and most Bi (III) is oxidized to be Bi (IV) which ensure long cycle life.

## Methods

### Synthesis of Bi_2_WO_6_ nanoparticles

The Bi_2_WO_6_ (BWO) nanoparticles were prepared via a solvothermal method. A typical process is as following: 0.97 g Bi(NO_3_)_3_·5H_2_O and 0.33 g Na_2_WO_4_·2H_2_O were dissolved in 40 ml ethylene alcohol, after being stirred for 6 h, the transparent solution was added into a 50 ml Teflon-lined autoclave up to 80% of the total volume. Then the autoclave was sealed in a stainless steel tank and heated at 180°C for 24 h, in which the heating rate is 1°C/min. Subsequently, the reactor was cooled to room temperature naturally, the products were washed by de-ionized water and alcohol in turn and then the BWO nanoparticles were obtained after being dried at 80°C in air.

### Synthesis of nano-Bi_2_WO_6_/reduced graphene oxide composites

Graphene oxide (GO) was prepared from natural graphite (99.95, 32 um) by a modified Hummers method^14^. 5 mg of GO, 95 mg of BWO nanoparticles and 30 ml of distilled water were mixed in a 50 ml Teflon-lined autoclave, and then followed by hydrothermal treatment of the mixture at 180°C for 24 hours. After the reaction, the products were harvested by centrifugation and washed with distilled water and ethanol for three times, and then dried in an oven at 80°C for 3 hours. The obtained Bi_2_WO_6_/reduced graphene oxide composite is labeled as BWO/RGO. As compared, reduced graphene oxide (RGO) was obtained under a similar hydrothermal progress without adding of BWO.

### Characterization

The crystal structures of the samples were characterized by using an X-ray diffractometer (XRD) (AXS D8 Advanced XRD, Germany) with Cu-Ka radiation. The microstructures were observed by using a high-resolution transmission electron microscope (HRTEM) (JEOL JEM 2010, Japan). X-ray photoelectron spectroscopy (XPS) measurements were made on a Kratos AXIS Ultra DLD (Kratos, Japan) spectrometer with a charge neutralizer to gain information on the chemical binding energy of the samples. The C 1s peak at 284.6 eV of the adventitious carbon was referenced to rectify the binding energies. Raman measurement was carried out using a Raman spectroscopy (HORIBA Jobin Yvon LabRAM HR, France). The power of the laser was 10 mW, and the laser excitation was 488 nm. Scans were taken on an extended range (100–3000 cm^−1^), and the exposure time was 2 s.

### The fabrication and electrochemical test of the materials

The working electrode was prepared by coating the active material onto nickel foam (1 cm × 1 cm) followed by pressing with a pressure of 0.5 tons. While the active material was the mixture of BWO or BWO/RGO, carbon black and PDFE binder (ratio 7:2:1). Before the coating, the mixture was ground in a mortar using a pestle, and the Grinding time was one hour. Then, the samples were uniformly distributed upon the Ni foam and allowed to dry in a vacuum oven for 2 hours. The end loading of active material for each electrode was 3 ~ 5 mg cm^−2^. At last, all of these electrodes were studied by Cyclic voltammetry (CV), Galvanostatic discharge and Electrochemical impedance spectroscopy (EIS) measurements on a commercial electrochemical workstation with three electrode system. Electrodes made from carbon materials worked as the working electrode, Ag/AgCl electrode as the reference electrode, and platinum network electrode as the counter electrode, the electrolyte is the 6 M KOH solution.

## Author Contributions

J.Z. proposed and designed the experiments. P.L., G.X., Z.L., X.W.,Y.W., L.Y. and X.T. carried out the experiments (synthesis and photocatalytic tests) and characterizations. J.Z. wrote the paper. Y.Z., H.W., E.Z. and Z.J. provide scientific advice. J.X. conducted the SEM and HRTEM measurements, respectively. All authors read and approved the final manuscript.

## Supplementary Material

Supplementary Information Supporting Information
