# Supplementary material for: Enhanced Performance of nano-Bi2WO6-Graphene as Pseudocapacitor Electrodes by Charge Transfer Channel
Source: Sci Rep. 2015 Feb 27;5:8624. doi: 10.1038/srep08624 (PMC4342564; doi:10.1038/srep08624)
Supplement: Supplementary Information — Supporting Information [file srep08624-s1.doc]

# Supporting Information for

# Enhanced Performance of nano-Bi_2_WO_6_-Graphene as [Pseudocapacitor](http://apps.webofknowledge.com/full_record.do?product=WOS&search_mode=CitingArticles&qid=6&SID=S1HC8A9XvZXwa6Jf3Bo&page=1&doc=11) Electrodes by [Charge Transfer Channel](http://apps.webofknowledge.com/full_record.do?product=WOS&search_mode=GeneralSearch&qid=5&SID=S1HC8A9XvZXwa6Jf3Bo&page=1&doc=7&cacheurlFromRightClick=no)

Jun Zhang^1^, *, Pengliang Liu^1^, Yupeng Zhang^4^, Guolong Xu^1^, Zhengda Lu^1^, Xiyu Wang^1^, Yan Wang^1^, Lingxia Yang^1^, Xi Tao^1^, Hongbo Wang^3^, Erpan Zhang^1^, Junhua Xi^1^ and Zhenguo Ji^1, 2^, *

^1^ *College of Materials and Environmental Engineering,* *Hangzhou Dianzi University, Hangzhou, 310018, People’s Republic of China*

^2^ *State Key Lab of Silicon Materials, Zhejiang University, Hangzhou, 310018, People’s Republic of China*

^3^ *College of Automation, Hangzhou Dianzi University, Hangzhou, 310018, People’s Republic of China*

^4^ *Department of Materials Engineering, Monash University, Victoria, 3800, Australia*

*** *Corresponding authors.* *Tel.: +86 0571 86878609; E-mail address:* [*zhangj@hdu.edu.cn*](mailto:zhangj@hdu.edu.cn) *and jizg2@zju.edu.cn*

## Supplementary Figures


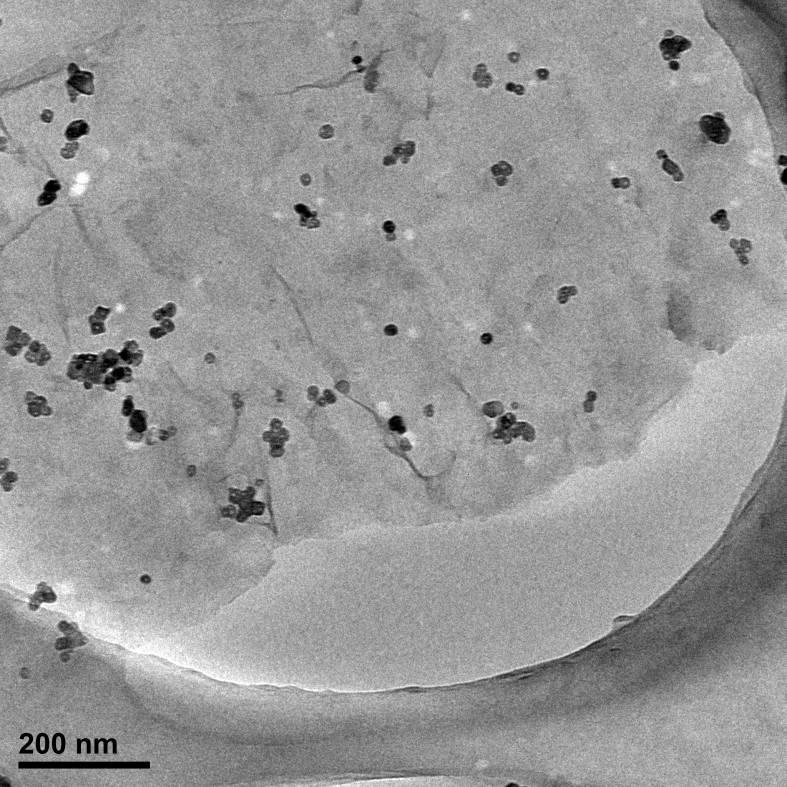


Fig. S1. TEM image of the composite obtained from reduced graphene oxide (RGO) and BWO nanoparticles


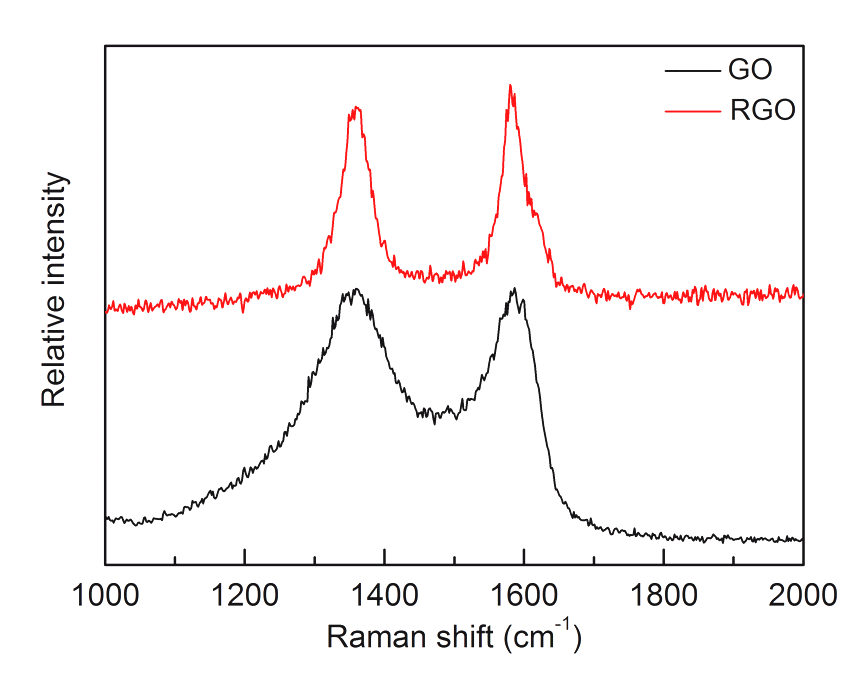


Fig. S2 Raman spectra of the GO synthesized chemical exfoliation and RGO from hydrothermal treatment


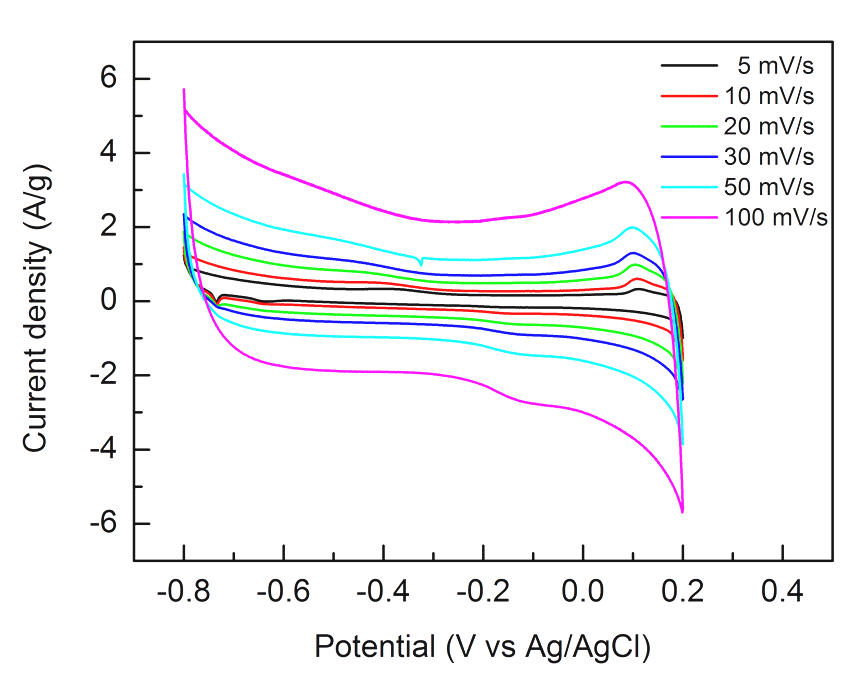


Fig. S3 CV curves of RGO at various scan rates
